# High-Throughput Analysis of Water-Soluble Forms of Choline and Related Metabolites in Human Milk by UPLC-MS/MS and Its Application

**DOI:** 10.3389/fnut.2020.604570

**Published:** 2021-02-05

**Authors:** Daniela Hampel, Setareh Shahab-Ferdows, Ngoc Nguyen, Gilberto Kac, Lindsay H. Allen

**Affiliations:** ^1^United States Department of Agriculture/Agricultural Research Service, Western Human Nutrition Research Center, Davis, CA, United States; ^2^Department of Nutrition, University of California, Davis, Davis, CA, United States; ^3^Nutrition Institute, Rio de Janeiro Federal University, Rio de Janeiro, Brazil

**Keywords:** human milk, water-soluble choline, metabolites, inter-relationships, UPLC-MS/MS

## Abstract

Choline and related metabolites are key factors in many metabolic processes, and insufficient supply can adversely affect reproduction and fetal development. Choline status is mainly regulated by intake, and human milk is the only choline source for exclusively breastfed infants. Further, maternal status, genotype, and phenotype, as well as infant outcomes, have been related to milk choline concentrations. In order to enable the rapid assessment of choline intake for exclusively breastfed infants and to further investigate the associations between milk choline and maternal and infant status and other outcomes, we have developed a simplified method for the simultaneous analysis of human milk choline, glycerophosphocholine, phosphocholine, and the less abundant related metabolites betaine, carnitine, creatinine, dimethylglycine (DMG), methionine, and trimethylamine *N*-oxide (TMAO) using ultraperformance liquid chromatography–*tandem* mass spectrometry (UPLC–MS/MS). These analytes have milk concentrations ranging over 3 orders of magnitude. Unlike other recently described LC-based methods, our approach does not require an ion-pairing reagent or high concentrations of solvent modifiers for successful analyte separation and thus avoid signal loss and potential permanent contamination. Milk samples (10 μl) were diluted (1:80) in water : methanol (1:4, v:v) and filtered prior to analysis with an optimized gradient of 0.1% propionic acid_aq_ and acetonitrile, allowing efficient separation and removal of contaminants. Recovery rates ranged from 108.0 to 130.9% (inter-day variation: 3.3–9.6%), and matrix effects (MEs) from 54.1 to 114.3%. MEs were greater for carnitine, creatinine, and TMAO at lower dilution (1:40, *p* < 0.035 for all), indicating concentration-dependent ion suppression. Milk from Brazilian women (2–8, 28–50, and 88–119 days postpartum, *n*_total_ = 53) revealed increasing concentration throughout lactation for glycerophosphocholine, DMG, and methionine, while carnitine decreased. Choline and phosphocholine were negatively correlated consistently at all three collection time intervals. The method is suitable for rapid analysis of human milk water-soluble forms of choline as well as previously not captured related metabolites with minimal sample volumes and preparation.

## Introduction

**Figure 1:**
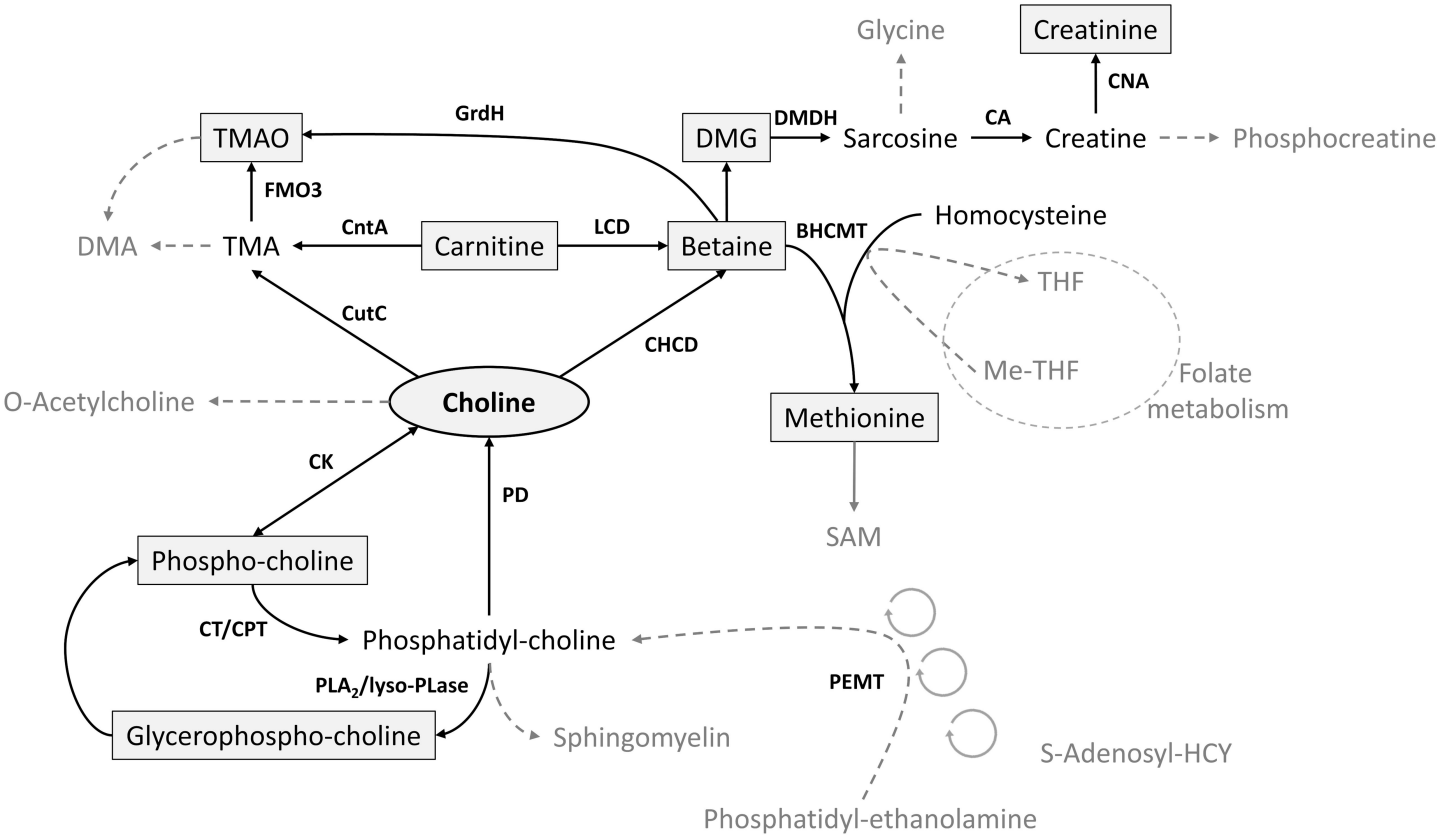
Key metabolic pathways of choline and its related metabolites (6–10). FMO3, flavin-containing monooxygenase; BHCMT, betaine-homocysteine methyl transferase; CA, creatinase; CAN, creatininase; CHCD, choline dihydrogenase; CK, choline kinase; CntA, carnitine monooxygenase; CPT, choline phosphotransferase; CT, cytidyltransferase; CutC, choline-TMA lyase; DMA, dimethylamine; DMDH, dimethylglycine dehydrogenase; DMG, dimethylglycine; GrdH, glycine-betaine reductase; LCD, l-carnitine dehydrogenase; lyso-PLase, lysophospholipase; PD, phospholipase; PEMT, phosphatidylethanolamine methyltransferase; PLA2, phospholipase A2; TMA, trimethylamine; TMAO, trimethylamine *N*-oxide.

Choline, an essential micronutrient, is a key factor in cellular maintenance and growth, including brain function, liver health, reproduction, and fetal and infant development (1–4). As a methyl donor, choline interacts with other metabolites of methyl group metabolism including betaine, methionine, dimethylglycine (DMG), glycine, and the folate pool (5) (Figure 1). Choline, betaine, and carnitine are precursors of trimethylamine *N*-oxide (TMAO), an osmolyte formed by the gut microbiota and associated with greater risk of cardiovascular disease, adverse thrombotic events, atherosclerosis, and colorectal cancer among postmenopausal women (11–13). Betaine is also a hepatic methyl donor and an important osmolyte to protect the cells of the renal medulla, and DMG in cord blood is positively correlated with birth weight (14).

Dietary choline can be obtained from animal source foods and also from various plants, such as nuts, legumes, or cruciferous vegetables (15). The liver is the main site for choline metabolism, where conversion into phosphatidylcholine (PC) occurs, via either the cytidine diphosphate (CDP)-choline pathway or the phosphatidylethanolamine *N*-methyltransferase (PEMT) pathway (16). The main biological functions of PC include membrane biosynthesis and myelination, lipid metabolism, cell division, or signaling molecules (15, 17). In lactation, maternal plasma choline is elevated, likely due to protecting intact choline, and not by upregulated *de novo* synthesis by the PEMT pathway as found in pregnancy (18). These reported higher maternal choline concentrations are thought to support increased infant needs in the first year of life, during which choline is a key factor for organ growth and membrane biosynthesis, and ensure efficient choline uptake by the brain and other tissues (15, 19).

Low choline status can affect reproduction and fetal development, leading to higher risk of birth defects, interference with neural tube closure, and poorer cognitive performance (4, 20). Choline status is principally maintained by dietary intake (5, 21, 22). Human milk is therefore the only choline supply for young infants if exclusively breastfed (EBF) as recommended by the World Health Organization (23). Moreover, higher milk choline concentrations in conjunction with higher milk lutein or DHA are related to better recognition memory in EBF infants (24), and milk choline and betaine are affected by maternal total choline intake and genotype (25). Thus, milk choline metabolites could be a useful proxy for infant and maternal status and genotype, as its sampling is less invasive than drawing blood, especially from infants, and there are no volume limitations.

Water-soluble free choline (Cho) represents ~90% of total milk choline, with phosphocholine (PCho) being the most abundant form, followed by glycerophosphocholine (GPCho), and Cho. The lipophilic forms phosphatidyl choline and sphingomyelin are only minor contributors (26–28). Throughout lactation, milk choline concentrations decline from colostrum to mature milk. Water-soluble forms of choline are considered milk constituents at all lactation stages, while the less abundant fat-soluble forms are associated with mature milk (15, 29). Choline uptake into the mammary epithelium is facilitated by a saturable, energy-dependent transporter system, or a non-saturable transport is activated with elevated maternal choline, where further choline metabolites can be derived (15).

Besides ^1^H-nuclear magnetic resonance (25, 30, 31), chromatographic techniques have emerged as the method of choice for choline analysis in human milk, employing liquid chromatography (LC) coupled with electrochemical detector or mass spectrometry (MS) (18, 26, 32–37) or gas chromatography (GC)–MS after a more complex sample preparation, including purification using LC with radiodetection, followed by hydrolysis of phosphorylated choline forms (27). Generally, sample preparation described in these reports included multiple sample preparation steps, including various extraction approaches, purification, hydrolysis, or derivatization. The more recently described LC–MS-based methods employ high concentrations of formic acid as a solvent modifier (34), which can reduce the signals of the target analytes (38), or achieve optimal analyte separation using trifluoroacetic acid (TFA), an ion-pairing reagent (26). Ion-pairing reagents, however, tend to remain in the LC system, columns, and MS source, which can lead to signal suppression and reduced analytical column performance (39). Thus, ion-pairing reagents should only be used with dedicated columns and equipment, which can greatly impact costs and instrument availability. The analysis of plasma or serum choline and some metabolites has been reported without ion-pairing reagents, but not to the same extent in human milk (11, 40, 41).

Here, we describe the first high-throughput method for simultaneous analysis of human milk Cho, PCho, GPCho, and previously not simultaneously analyzed related metabolites betaine, carnitine, creatinine, DMG, methionine, and TMAO (Supplementary Figure 1) using ultraperformance LC (UPLC)–MS/MS, requiring only minimal sample volume and preparation, without the need for ion-pairing reagents or high concentration of solvent modifiers. The optimized method was used to analyze milk samples from Brazilian mothers collected at different stages of lactation.

## Materials and Methods

### Chemicals and Materials

Choline chloride, l-α-PCho, betaine, PCho-(trimethyl-d9) chloride, l-methionine-(methyl-d3), and creatinine were purchased from Millipore-Sigma (Burlington, MA, USA). TMAO dihydrate, l-methionine, *N, N*-dimethylglycine hydrochloride, l(–)-carnitine, LC–MS-grade acetonitrile (ACN), LC–MS-grade methanol (MeOH), LC-MS-grade 2-propanol (IPA), LC–MS-grade water, and propionic acid were obtained from Fisher Scientific (Hampton, NH, USA), and l-α-glycerophosphoryl (choline-d9) was obtained from Avanti Polar Lipids (Alabasta, AL, USA). Choline chloride (trimethyl-d9), TMAO (d9), creatinine (*N*-methyl-d3), l-carnitine (trimethyl-d9), and betaine-d11 were purchased from Cambridge Isotope Laboratories (Tewksbury, MA, USA), and *sn*-glycero-3-PCho was purchased from AK Scientific (Union City, CA, USA).

Two-milliliter amber, screw-top vials, deactivated 150 μl glass inserts with plastic springs, and pre-slit PTFE/silicone screw caps were obtained from Waters (Milford, MA, USA), and 1.5 amber snap-cap centrifuge tubes and Ultrafree®-MC-VV centrifugal filters (Durapore® PVDF) 0.1 μm were purchased from Fisher Scientific (Waltham, MA, USA).

### UPLC–MS/MS

Method development and analyses were carried out using an ACQUITY I-Class UPLC (Waters, Milford, MA, USA) coupled to a Triple Quad 4500 mass spectrometer (Sciex, Framingham, MA, USA). Optimal results were achieved using a Luna Silica (2) column, 100 × 2 mm, 3 μm, protected by a SecurityGuard (silica, 2 mm, 3 μm; Phenomenex, Torrance, CA, USA) held at 40°C. Ten microliters of the sample extract were injected and separated using a solvent gradient of 0.1% aqueous propionic acid (A) and ACN (B) at 0.275 ml/min as follows: 0–1 min, 60% A; 3 min, 90% A; 4 min, 90% A; and 4.2–7.5 min, 60%. Samples were maintained at 8°C throughout the analysis. After each sample injection, a needle wash [weak: water/MeOH/IPA (7/2/1, v/v/v), strong: ACN/MeOH_50/50_/water (8/2, v/v)] was performed; seal wash was performed using the weak wash.

Analyte optimization was done by infusion of a 100 μg/L solution for each compound in water: MeOH (1:1, v:v) at a flow rate of 7 μl/min and positive ion mode electrospray ionization (ESI). The analytes were detected with multiple reaction monitoring (MRM, Table 1). Curtain gas (20 psi), CAD gas (6 psi), ion spray voltage (4,500 V), turbo gas temperature (550°C), ion source gases 1 and 2 (40 psi), entrance potential (10 V), and dwell time (20 ms) were identical for all analytes in the positive mode. An integrated two-position switch valve was used to regulate effluent flow into the MS (1.45–6 min) during the analytical run, which allowed the removal of the early eluting endogenous lactose. The efficiency of this removal was further monitored using MRM in negative ion mode electrospray ionization: ion spray voltage (−4,500 V) and entrance potential (−10 V). The switch time of 6 min was chosen to allow sufficient flow from the valve to the MS inlet, reducing the potential build-up of the sample matrix in the flow path to the MS.

**Table 1:** Multiple reaction monitoring (MRM) parameters for target analytes.

**Analyte**	**t_**R**_ (min)**	**Q1 (m/z)**	**Q3 (m/z)**	**DCP (V)**	**CE (eV)**	**CXP (V)**
Choline	4.24	104.1	60.1	40	22	8
PCho	2.72	184.0	86.0	40	24	8
GPCho	1.97	258.0	124.9	65	33	8
Betaine	1.85	117.9	58.0	61	39	6
DMG	1.57	103.9	58.0	1	17	16
Carnitine	3.35	161.9	58.0	46	57	6
Creatinine	3.14	113.9	85.9	6	15	8
Methionine	1.49	149.9	103.9	11	13	6
TMAO	3.82	76.0	58.1	46	35	6
**Internal standards**						
Choline-d9	4.23	113.0	45.0	40	22	8
PCho-d9	2.71	193.0	95.0	40	24	8
GPCho-d9	1.96	267.1	125.0	65	33	8
Betaine-d11	1.85	129.0	66.0	61	25	6
Carnitine-d9	3.34	171.0	66.0	26	59	6
Creatinine-d3	3.13	116.9	89.0	51	15	8
Methionine-d3	1.48	152.9	107.0	11	15	8
TMAO-d9	3.81	85.0	66.1	61	41	6
Lactose	<1.45	341.2	161.2	−30	−30	−8

*CE, collision energy; CXP, collision cell exit potential; DCP, declustering potential; DMG, dimethylglycine; GPCho, glycerophosphocholine; PCho, phosphocholine; TMAO, trimethylamine N-oxide. Lactose MRM was used to monitor lactose removal efficiency*.

### Standard Preparation

All analytes (external standards) and internal standards (54.5–112 μg/L) were prepared in LC–MS-grade water: methanol (80:20, v:v) and stored in amber glass vials at −80°C. A master mix was prepared in sample diluent (water: MeOH 1:4, v:v) containing 2,500 μg/L Cho, 5,000 μg/L GPCho, 7,500 μg/L PCho, and 500 μg/L for the remaining analytes (betaine, carnitine, creatinine, DMG, methionine, and TMAO). The calibration curve was prepared by further diluting the master mix in sample diluent and by adding 10 μg of a prepared internal standard (IS) mix (2 mg/L for GPCho and PCho and 1 mg/L for all remaining analytes). Besides being added to the calibration curve, the same amount of IS mix was added to each blank and sample.

### Sample Preparation

Five to 10 μl of whole human milk was diluted [dilution factor (dF) = 40 or 80 in sample diluent], mixed, and centrifuged (14,000 rpm, 10 min, 4°C). Ninety microliters of the diluted samples were transferred into the centrifugal filters and combined with 10 μl of the IS mix. The sample extracts were briefly mixed prior to filtration in a second centrifugation step (6,000 × g, 4 min, 4°C). The filtered samples were transferred into 2-ml amber LC vials equipped with 150-μl glass inserts for analysis by UPLC–MS/MS as described above.

### Human Milk Samples

A human milk pool from a healthy donor in the Sacramento (CA, USA) area was used for method development, validation purposes, and quality control (QC) during sample analysis. For a quick comparison of recovery rates for the primary analytes Cho, PCho, and GPCho, additional milk samples from a single donor in the Davis (CA) area (MD2) and a milk pool from mothers in the Vancouver (BC, Canada) area (MD3) were used for recovery experiments as described. The validated method was used to analyze convenient milk samples (*n* = 53) from healthy Brazilian mothers (18–40 years) who were part of a prospective cohort study conducted in a Public Health Care Center in Rio de Janeiro (42). Milk samples were collected at different stages of lactation (group A: 2–8 days, *n* = 11; B: 28–50 days, *n* = 18; and C: 88–119 days, *n* = 24). Nine women provided samples at two time intervals (A/B, *n* = 2; B/C, *n* = 5; A/C, *n* = 2), and five women at all three time intervals. All samples were shipped on ice to the USDA/ARS Western Human Nutrition Research Center in Davis, CA, USA, and stored immediately at −80°C with light exclusion until use.

### Method Validation, Linearity, Stability, and Matrix Effects

The validation of the developed method was carried out following Food and Drug Administration guidelines (43). Standard addition experiments at three different levels were carried out in five replicates on three different days using pooled human milk with unknown analyte concentrations. The master mix was used to spike the diluted milk samples with 25, 50, and 75 μg/L (5, 10, and 15 μl) of betaine, carnitine, creatinine, DMG, methionine, and TMAO. Spiking levels (L1, L2, and L3) were 125, 250, and 375 μg/L for Cho; 250, 500, and 750 μg/L for GPCho, and 375, 750, and 1,125 μg/L for PCho. With each standard addition experiment, five non-spiked milk samples were analyzed. To account for the volumetric changes due to the different spiking volumes, all samples were adjusted to the sample volume using the diluent as needed. For a quick comparison of recovery rates of the main analytes Cho, PCho, and GPCho, two additional milk samples were subjected to the described the standard addition.

Analyte recovery was estimated using the following equation:

(1) $$\begin{array}{r} \text{Recovery}\left(\%\right)=\frac{\left({\text{C}}_{\text{measured}}-{\text{C}}_{\text{endogenous}}\right)\;*\;100}{{\text{C}}_{\text{added}}} \end{array}$$

where *C*_measured_ denoted the measured concentration of the spiked sample, *C*_endogenous_ the measured concentration of the non-spiked sample, and *C*_added_ the theoretically added concentration.

Signal-to-noise (S/N) ratios were examined in three analytical runs for the lowest standard curve concentrations for each target analyte. Limit of detection (LOD) was achieved for S/N > 3, and limit of quantitation (LOQ) for S/N > 10.

Linearity and reproducibility of standard curves were evaluated by inter-day variability (mean and CV) of slopes, coefficients of determinations (*r*), coefficients of correlations (*r*^2^), and back-calculation to the nominal standard concentrations using eight standard curves from eight different days over 1 month. Analyte stability in milk was monitored in the pooled human milk samples stored at −80°C and prepared in five runs over 2 months (*n* = 15).

The matrix effect (ME) was determined using the isotopically labeled IS as described by Matuszewski et al. (44). The neat standards (set 1) at 100 μg/L were prepared in diluent (*n* = 9), while milk samples at two different dilutions (dF 40 and 80) were used to prepare set 2, by adding the IS after at the end of the sample preparation. Each dilution was analyzed in two separate analyses, for a total of four sets.

ME was calculated using the following equation:

(2) $$\begin{array}{r} \text{ME}\left(\%\right)=\frac{\text{set}2\;*\;100}{\text{set}1} \end{array}$$

### Standard Curve and Quality Control

For each validation experiment and for sample analyses, an eight-point calibration curve (betaine, carnitine, creatinine, DMG, methionine, TMAO: 1–500 μg/L; Cho: 5–2,500 μg/L; GPCho: 10–5,000 μg/L; PCho: 15–7,500 μg/L) and a reagent blank (diluent) were prepared in diluent. Since the calibration curve was not diluted, the true analyte concentrations were calculated by adjusting the concentrations obtained from the sample extracts by the dilution factor (dF = 80 or 40). The validated pooled human milk was used as the quality control (QC) with each sample batch and analyzed every 24 samples. A typical batch consisted of a blank, calibration curve, four QCs, and 82 samples for a total of 95 samples when using two of the UPLC 2-ml 48-vial holder racks.

Quantification was carried out by area ratio response to the respective stable-isotope IS to account for extraction efficiency, volumetric changes, and ion suppression. Since no IS for DMG was available in the laboratory, DMG was quantified using betaine-d11.

### Data and Statistical Analysis

MultiQuant Software (version 3.03, Sciex, Framingham, MA, USA) was applied for data processing, analysis, and S/N ratio evaluation. Calculations for method validation (e.g., mean, SD, CV, ME, and analyte recovery) were carried out using Excel 2016 (Microsoft, Redmond, WA, USA). RStudio (version 1.3.959, R Foundation for Statistical Computing, Vienna, Austria) in conjunction with R Statistical Software (version 4.0.0) was used for statistical analysis and correlation maps. Student's *t*-test was used to examine difference in MEs by dilution factor. Data normality was evaluated using the Shapiro–Wilk test. Significant differences in analyte concentrations in milk from Brazilian mothers based on stage of lactation were examined by Kruskal–Wallis test followed by Dunn's test adjusted for multiple comparison by Bonferroni. The Kruskal–Wallis test was also used to test for difference in recovery rates among different milk samples. Spearman's rank correlation was used to evaluate associations among the target analytes. Correlation strength was classified as follows: weak (ρ <0.3), moderate (ρ = 0.3–0.5), good (ρ = 0.5–0.7), and strong (ρ > 0.7) (45). *p*-values < 0.05 were considered significant.

## Results

### Chromatography

**Figure 2:**
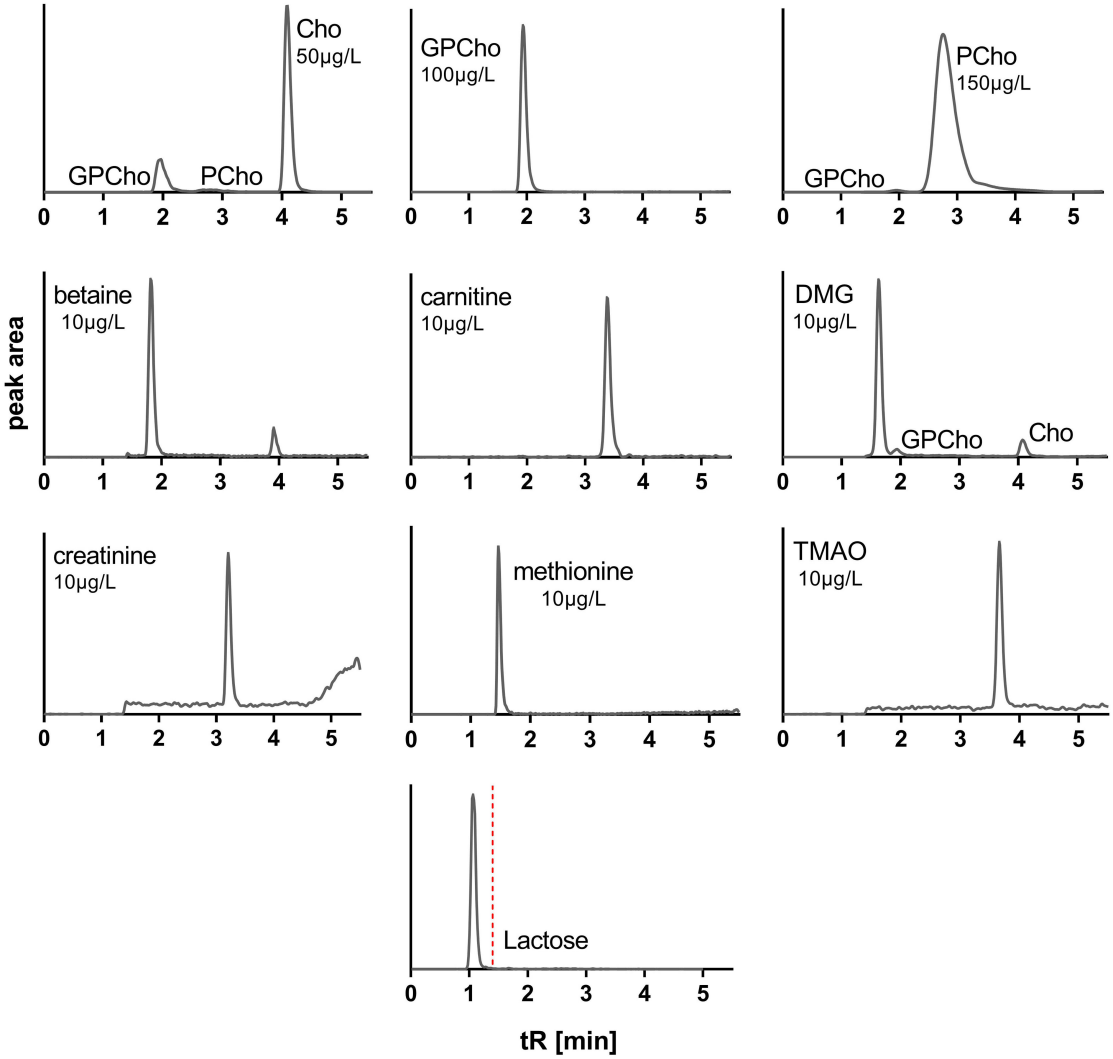
Extracted ion chromatograms of standards of water-soluble forms of choline and related metabolites and lactose. Cho, choline; GPCho, glycerophosphocholine; PCho, phosphocholine; DMG, dimethylglycine; TMAO, trimethylamine *N*-oxide; tR, retention time. MRM cross talk between analytes is observable but does not interfere.

During method development, additional columns, solvent mixtures, and gradients in normal and reversed-phase modes were tested, including Waters ACQUITY UPLC HSS T3 (1.8 μ, 2.1 × 100 mm), ACQUITY BEH Amide (1.7 μ, 2.1 × 50 mm) columns, and Phenomenex Kinetex HILIC (1.7 μ, 2.1 × 100 mm) column. Solvent systems tested included aqueous buffers (10 mM ammonium formate, 0.1% acetic acid, or 0.1% propionic acid) and MeOH or ACN with and without modifiers (0.1% acetic or propionic acid). For a full list of tested columns and solvents, see Supplementary Table 1. The above-described normal-phase conditions using the Luna Silica (2) column, 0.1% aqueous propionic acid (solvent A), and ACN (solvent B) provided optimized separation to avoid cross talk or carryover interferences and peak shapes for all target analytes, while also allowing chromatographic removal of endogenous lactose to avoid MS contamination (Figure 2).

### Method Validation, Linearity, MEs

All analytes were directly extractable using water: MeOH (1:4, v:v). No additional reagents were necessary for this application. Five and 10 μL sample volumes were used successfully during sample preparation, but we opted for the larger volume to reduce possible pipetting errors at minute volumes.

Since there is no certified human milk reference material, analyte recovery was determined using pooled human milk with unknown concentrations of the analytes using the standard addition method. Recoveries ranged between 108.0 and 130.9% for all target analytes over the three levels of standard addition. Inter-day variations ranged between 3.3 and 9.0% (Table 2), intra-day variations from 1.7 to 8.8% for all analytes and days. Comparable recovery rates for Cho, PCho, and GPCho (*p* ≥ 0.1 for all) were found in milk from additional donors (MD2 and MD3, Supplementary Table 2).

**Table 2:** Target analyte concentrations (±SD) and recovery rates (CV) in pooled human milk.

**Analyte**	**C (mg/L)**	**SD**	**L1 (%)**	**CV**	**L2 (%)**	**CV**	**L3 (%)**	**CV**	**Overall (%)**	**CV**
Choline	29.5	±0.80	105.2	(3.4)	110.6	(1.8)	108.8	(3.3)	108.2	(3.3)
PCho	100	±3.2	115.7	(2.5)	107.0	(3.3)	108.2	(5.3)	110.3	(4.7)
GPCho	241	±7.2	116.0	(4.4)	113.6	(5.3)	109.3	(2.6)	113.0	(4.6)
Betaine	0.47	±0.03	124.5	(7.5)	120.8	(8.9)	122.2	(6.2)	122.5	(6.7)
Carnitine	6.94	±0.25	133.5	(9.0)	132.6	(10.1)	126.5	(10.7)	130.9	(9.0)
Creatinine	4.14	±0.14	134.3	(8.7)	120.8	(9.7)	120.3	(9.0)	125.1	(9.6)
DMG	0.32	±0.01	121.9	(10.2)	118.8	(13.1)	117.6	(8.9)	119.4	(8.6)
Methionine	0.36	±0.01	119.8	(6.5)	118.6	(9.0)	117.4	(7.6)	118.6	(6.8)
TMAO	0.16	±0.01	109.0	(3.2)	106.8	(6.3)	108,3	(7.4)	108.0	(5.2)

*Recoveries were determined in four replicates on three different days. C, concentration; CV, coefficient of variation; GPCho, glycerophosphocholine; DMG, dimethylglycine; L1, analyte recovery of level 1 standard addition; L2, level 2 analyte recovery of standard addition; L3, analyte recovery of level 3 standard addition; PCho, phosphocholine; SD, standard deviation; TMAO, trimethylamine N-oxide. Standard addition concentrations for L1–L3 were as follows: Cho 125, 250, and 375 μg/L; GPCho 250, 500, 750 μg/L; PCho 375, 750, and 1,125 μg/L; betaine, carnitine, creatinine, DMG, methionine, and TMAO with 25, 50, and 75 μg/L*.

*SD and CV and represent inter-day variations of analyte concentrations and recovery at each level and overall recovery*.

A 1:80 sample dilution revealed MEs between 75 and 115% for most analytes (Table 3); carnitine-d9 showed an ME of 54.1%. The lower sample dilution (1:40) resulted in significantly lower ME for carnitine-d9, creatinine-d3, and TMAO-d9 (*p* ≤ 0.034 for all). No data were available for DMG since an isotopically labeled DMG was not available.

**Table 3:** Matrix effects using stable-isotope IS of target analytes in human milk.

**Analyte**	**ME dF40 (%)**	**CV**	**ME dF80 (%)**	**CV**	***p*-value**
Choline-d9	88.9	(7.3)	96.2	(3.3)	0.18
PCho-d9	103.4	(3.5)	114.3	(7.6)	0.14
GPCho-d9	90.5	(5.7)	88.1	(6.6)	0.63
Betaine-d11	109.3	(1.9)	104.2	(5.6)	0.24
Carnitine-d9	30.3	(8.1)	54.1	(13.6)	**0.034**
Creatinine-d3	68.6	(2.3)	89.8	(4.3)	**0.014**
Methionine-d3	73.5	(11.6)	90.5	(5.7)	0.06
TMAO-d9	97.2	(3.6)	107.0	(1.6)	**0.022**

*Matrix effects were determined on three separate days for each dilution (n = 9 for each sample set and day for a total n = 27). Means are presented. Student's t-test was used to examine significant difference in matrix effects. Bold font indicates statistical significance (p < 0.05). CV, coefficient of variation; GPCho, glycerophosphocholine; IS, internal standard; ME, matrix effect; PCho, phosphocholine; TMAO, trimethylamine N-oxide. At the time of the validation, an IS was unavailable for dimethyl glycine; thus, ME was not evaluated for this analyte*.

The standard curves (*n* = 8) over 1 month showed good inter-day linearity. However, the initial chosen standard curve concentration range for Cho up to 2,500 μg/L revealed a lower trend line coefficient of correlation [$r_{\text{mean}\left(\text{CV}\right)}^{2}$ = 0.990 (0.4%)] and a mean slope of 0.85 (CV: 3.8%). By excluding the highest concentrations, *r*^2^ and slope improved (*r*^2^ = 0.997, *m* = 1.02; Table 4). The adjusted use of standard levels for Cho allowed for average trend line slopes for all analytes ranging from 0.96 to 1.02 (SD: ±0.007 to ±0.024). Coefficients of determinations (*r*) were above 0.997 (CV < 0.25%), and coefficients of correlation (*r*^2^) above 0.995 (CV < 0.50%; Supplementary Figure 2). Differences to the nominal concentrations varied between 94.9 and 101.3% for all analytes. The lowest standard curve concentrations for all analytes revealed a S/N > 10 (S/N for all: 11–404; Table 4).

**Table 4:** Signal-to-noise ratios (mean ± SD) of the lowest standard curve concentrations and goodness of standard curves.

**Analyte**	**C (μg/L)**	**S/N (mean)**	**SD**	**Δ_nom.conc._ (mean. %)**	**SD**	**Slope (mean)**	**SD**	***r*^**2**^**
Choline	5.0	127	±19	99.5	±0.14	1.02	±0.023	0.997
PCho	15	404	±42	95.9	±0.13	1.00	±0.018	0.998
GPCho	10	146	±14	94.9	±0.12	1.00	±0.008	>0.999
Betaine	1.0	11	±1.6	98.7	±0.01	0.98	±0.023	0.995
Carnitine	1.0	34	±2.2	98.4	±0.12	1.01	±0.007	>0.999
Creatinine	1.0	13	±0.8	95.2	±0.13	1.00	±0.010	0.999
DMG	1.0	163	±21	100.0	±0.08	0.96	±0.016	0.998
Methionine	1.0	213	±16	96.4	±0.11	0.99	±0.010	0.999
TMAO	1.0	38	±4.3	101.3	±0.08	0.97	±0.020	0.996

*Signal-to-noise ratios were determined in replicates of three. Goodness of the standard curves was evaluated using eight curves acquired over 1 month. DMG, dimethylglycine; GPCho, glycerophosphocholine; PCho, phosphocholine; r^2^, coefficient of correlation of the standard curve; SD, standard deviation; S/N, signal-to-noise ratio; TMAO, trimethylamine N-oxide; Δ_nom.conc._, difference among nominal concertation of standards*.

**Figure 3:**
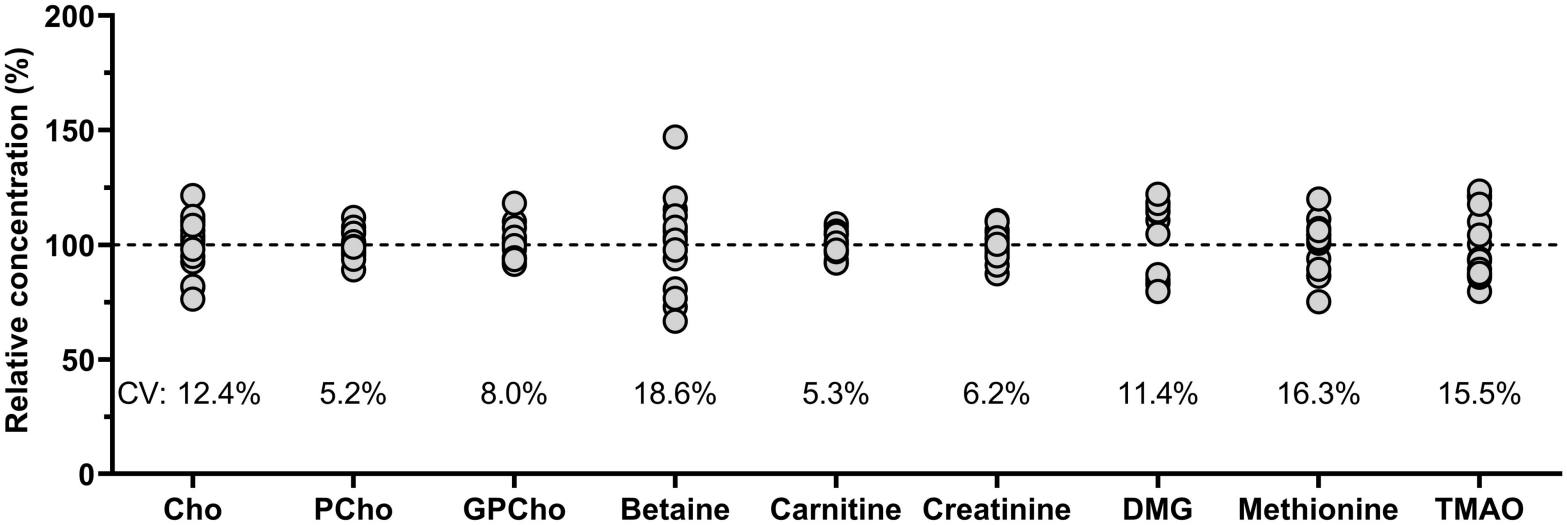
Relative concentrations (%) based on overall analyte mean of pooled human milk analyzed on five different days over 2 months by two different researchers (*n* = 15). Cho, choline; CV, coefficient of variation; Cho, choline; GPCho, glycerophosphocholine; PCho, phosphocholine; DMG, dimethylglycine; TMAO, trimethylamine *N*-oxide.

Comparing the measured QC sample concentrations in five runs over 2 months, conducted by two researchers showed high stability with inter-day variations well below 10% for most analytes (Figure 3). Betaine revealed the greatest variation but was still within run acceptance criteria (43).

### Analysis of Brazilian Human Milk Samples at 0–3 Months of Lactation

Colostrum and milk samples from Brazilian mothers revealed significant differences in concentrations depending on the time of collection (groups A, B, and C) for some metabolites (Table 5). GPCho, DMG, and methionine concentrations increased from colostrum to 3 months postpartum, while Cho and PCho remained steady. Although the Kruskal–Wallis test indicated significant differences for PCho concentrations, these differences were no longer found after further investigation using Dunn's test. Carnitine revealed a nonsignificant *p*-value, but the pairwise comparison indicated a significant decrease in concentration between groups A and C.

**Table 5:** Median concentrations (IQR) of choline and related metabolites in colostrum and milk from apparently healthy Brazilian mothers.

**Analyte**	**A (mg/L)**	**IQR**	**B (mg/L)**	**IQR**	**C (mg/L)**	**IQR**	***p*-value^1^**
Choline	12.9	(10.0, 16.3)	12.4	(9.8, 20.3)	15.7	(11.7, 21.1)	0.31
PCho	222	(125, 241)	191	(150, 229)	148	(132, 181)	**0.037**
GPCho	34.6^a^	(22.5, 54.9)	82.6^b^	(58.3, 104)	78.1^b^	(66.2, 106)	**0.006**
Betaine	0.39	(0.30, 0.48)	0.42	(0.23, 0.59)	0.38	(0.19, 0.49)	0.80
Carnitine	5.04^ab^	(2.25, 5.34)	4.53^a^	(3.85, 5.73)	3.59^b^	(3.33, 4.10)	**0.051**
Creatinine	3.56	(3.26, 4.03)	3.34	(3.00, 4.25)	3.36	(2.87, 3.65)	0.20
DMG	0.22^a^	(0.14, 0.32)	0.38^a^	(0.27, 0.48)	0.53^b^	(0.44, 0.60)	**<0.001**
Methionine	0.37^a^	(0.28, 0.52)	0.58^ab^	(0.46, 0.87)	0.70^b^	(0.61, 0.91)	**0.014**
TMAO	0.15	(0.09, 0.23)	0.09	(0.06, 0.19)	0.09	(0.06, 0.19)	0.28

*A: colostrum samples collected 2–8 days pp (n = 11); B: samples collected 28–50 days pp (n = 18); C: samples collected 88–119 days pp (n = 24); DMG, dimethylglycine; GPCho, glycerophosphocholine; IQR, interquartile range; PCho, phosphocholine; pp, postpartum; SD, standard deviation; TMAO, trimethylamine N-oxide*.

1 *Overall significant differences (p < 0.05) were examined by Kruskal–Wallis test. Dunn's tests adjusted for multiple comparison by Bonferroni when Kruskal–Wallis indicated significant differences. Significant differences (Dunn's test) are indicated by different superscript letters*.

**Figure 4:**
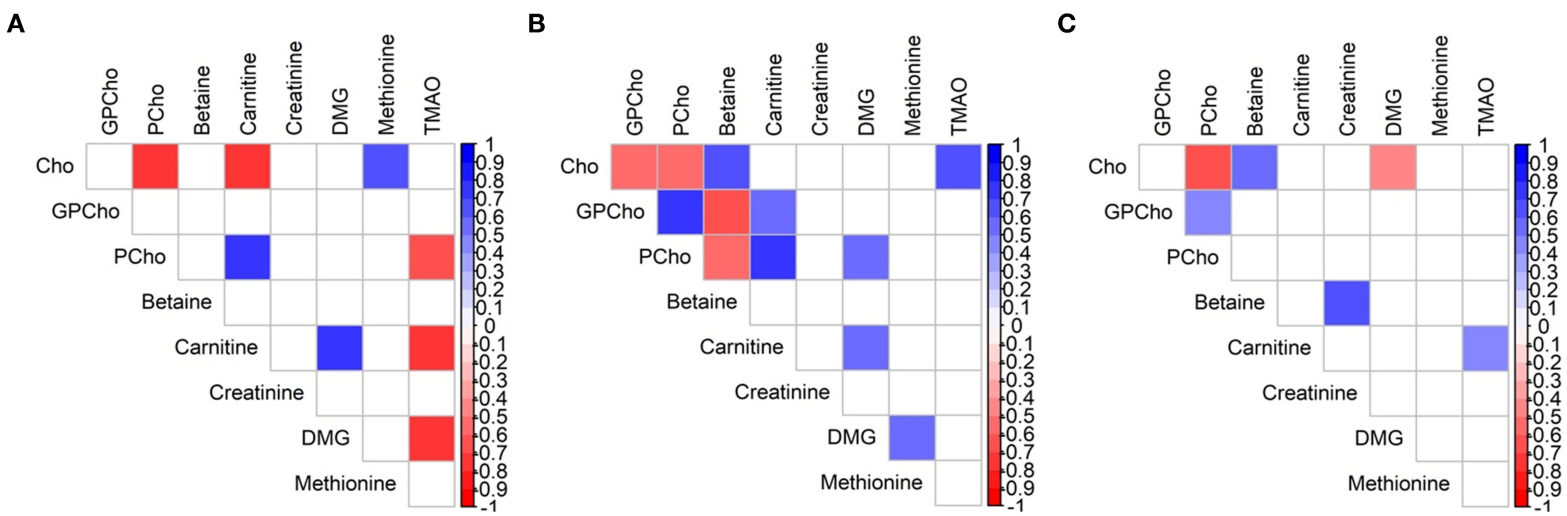
Spearman rank correlation map of water-soluble choline and related metabolites in milk of Brazilian mothers at (**A**) group A (*n* = 11), (**B**) group B (*n* = 18), and (**C**) group C (*n* = 24). Only significant correlations are indicated (*p* < 0.05). Cho, choline; DMG, dimethylglycine; GPCho, glycerophosphocholine; PCho, phosphocholine; TMAO, trimethylamine *N*-oxide.

All significant associations between the analytes were moderate to strong, regardless of group assignment and time interval, but correlation patterns changed over time (Figure 4). The negative correlation between Cho and PCho was the only one which was conserved throughout the three groups (ρ = −0.54 to −0.71, *p* < 0.018 for all). Consistent correlations between two groups were found for carnitine/PCho, carnitine/DMG (A/B), Cho/betaine, and GPCho/PCho (B/C; ρ = 0.45–0.77, *p* < 0.03 for all). While carnitine and TMAO were strongly negatively associated in group A, this relationship inversed in group C (ρ = −0.71 vs. 0.43, *p* < 0.04 for all, Supplementary Table 3).

## Discussion

### Chromatography and Sample Preparation

Among all tested LC columns, solvent systems, and gradients, the described conditions emerged as the most suitable for analyzing all target analytes. Given the structural similarities of some target analytes (Supplementary Figure 1), the use of similar MRM transitions was unavoidable, e.g., Cho, PCho, DMG, and GPCho. However, the optimized conditions fully separated all affected analytes, avoiding cross talk interferences (Figure 2).

Water-soluble vitamins in human milk are typically analyzed after removal of protein and lipophilic milk components to reduce matrix interferences (46, 47). MeOH effectively precipitates human milk protein while conserving the water-soluble target analytes in the sample extract (46). Although the samples here were highly diluted, therefore reducing potential matrix interferences, for our diluent, we chose a water/MeOH ratio commonly used for protein precipitation (48) to obtain a cleaner sample extract. While this minimalistic sample preparation did not remove the endogenous lactose, a major interference and MS contaminant, the optimized chromatographic conditions fully separated lactose from the target analytes, which were removed by a post-column, integrated two-position switch valve. Moreover, the sample dilution of 1:80 reduced lactose concentrations (~7%) below 0.1%, simplifying the lactose removal by lowering its abundance while still enabling the analysis of the highly abundant water-soluble choline forms simultaneously with the low-level metabolites.

### Method Validation and MEs

Recovery rates for all analytes were above 100%, possibly due to the minimal sample preparation, which is accompanied by lower matrix removal efficiency. Matrix components which remain in the sample extract used for analysis could affect the recovery when co-eluting with the target analytes. This interference could be occurring in particular for analytes revealing recovery rates consistently above 120%, such as betaine, carnitine, and creatinine. Since matrix components are independent of the spiking level, such possible effects should be consistent across the different levels of standard addition, as observed here. Furthermore, the IS mix was added after the dilution and centrifugation step due to the otherwise considerable increased required concentrations of IS and the limited availability in the laboratory. Thus, the IS was not subjected to the described initial sample dilution, which could contribute to the higher recovery rates. Nevertheless, analyte recoveries were not only consistent across the different standard addition levels and over time but, albeit only tested for Cho, PCho, and GPCho, also across milk from different donors (QC, MD2, and MD3). These results emphasize the importance of standard addition experiments during method validation to enable accurate measurements, particularly when no certified reference material is available.

MEs are a source for ion suppression and enhancement and may affect detection, accuracy, and precision. Co-elution of matrix components, which were not removed during sample preparation, has been recognized as a main ME contributor (49). MEs for most analytes here ranged around 75–115% with no significant difference in ME between the 1:40 and 1:80 diluted samples, suggesting only minor ion suppression or enhancement effects, independent of sample dilution. However, carnitine and creatinine revealed considerable ion suppression with significantly improved MEs in the more highly diluted samples. The continued noticeable ion suppression for carnitine did not interfere with quantification in the human milk matrix. Phospholipids have been identified as a significant source of matrix interferences (50), and their removal could improve recoveries and MEs. Since fat removal was sacrificed for a more simplified sample preparation procedure, future applications, e.g., in the 96-well plate format, could include a phospholipid removal step, which will not considerably increase sample preparation time but could be beneficial for MEs and analyte recoveries.

### LOQ, Standard Curve, and Analyte Stability

The S/N ratios of all lowest standard curve concentrations were above LOQ (>10), and median concentrations of all metabolites in the pooled human milk and in the Brazilian milk samples were above the lowest standard curve concentrations. TMAO concentrations in the samples were the closest to the low end of the standard curve, albeit within range. However, with an average S/N ratio of 38, the TMAO curve can be extended below the currently chosen lowest concentrations if needed. This was true for all other target analytes but betaine. Betaine concentrations in our experiments were on average about five-fold higher than the lowest standard, falling well into the chosen standard curve range.

The initial trend line slope of 0.85 for Cho obtained when all prepared standard levels were included indicated some bias between nominal and measured concentrations, likely due to extending the standard curve past the limit of linearity. By excluding the high standard, we preserved a sufficient number of standard levels (43), as well as the needed quantitation range.

All analytes in the QC milk sample analyzed over 2 months clustered around the nominal concentrations as established by the standard addition experiments. The greatest variation was found for betaine, which could point toward a potentially more sensitive metabolite, but since these analyses were conducted by two different researchers, additional between-person variation could have been introduced.

### Application to Human Milk From Brazilian Mothers

For our pilot study of 53 milk samples from Brazilian mothers, we found declining concentrations with duration of lactation for PCho and carnitine, while GPCho, DMG, and methionine increased. Analyzing different forms of choline in milk from Turkish women at different lactation stages (0–2 and 12–180 days), Ilcol et al. (35) reported increasing Cho and GPCho concentrations and no change in PCho, while Holmes et al. (31) reported significantly increasing concentration in milk from English mothers for all three water-soluble choline forms from 2–6 to 7–22 days. Little information is available about the longitudinal changes of these milk choline metabolites throughout lactation; thus, our data add to the knowledge base on human milk composition.

Comparing our group A choline results to other published water-soluble choline concentrations in colostrum collected in England and Turkey (31, 35), we found comparable concentrations to Cho and GPCho, but two-fold to five-fold higher PCho concentrations. This trend for Cho and PCho was also observable when comparing our results for groups B and C to milk samples collected up to 28 weeks of lactation in Canada, Cambodia, the USA, and the aforementioned studies in Turkey and England, but GPCho concentrations found in our sample set were about 1.2–1.8-fold lower (31, 35–37, 51). While betaine concentrations in our milk samples were comparable to concentrations found in samples from Germany (34), they were about 10-fold lower than the reported values from US women (51). Concentrations found here were lower for carnitine (1.3–2.6-fold), creatinine (1.3–1.8-fold), and DMG (1.6–3.8-fold) than reported literature values from studies conducted in Germany (34, 52, 53). Methionine concentration in the literature varied considerably by geographic origin, with lowest values reported from German milk samples (0.03 mg/L) (34) and highest in a study conducted in Italy (1.3 mg/L) (54), while concentrations reported from a study in Bangladesh were similar to our results (55). TMAO was not detected in milk from German mothers (34), which is in contrast to our findings. These comparisons illustrate the wide variations in concentrations of water-soluble forms of choline and related metabolites, which may be affected by factors such as geographic origin, stage of lactation, maternal diets, and genotypes or phenotypes.

The found differences in the correlation patterns in all three groups (A, B, and C) are another indication for concentration changes over the duration of lactation. The only conserved association throughout all lactation stages was observed for Cho and PCho, which has also been previously reported, albeit as a weak association, by Gay et al. (56). The continuous correlations between Cho and betaine and between GPCho and PCho in groups B and C were also in agreement with the previous report (56), while Maas et al. (34) did not find a relationship between Cho and betaine or between Cho and carnitine. Consistent correlations between two groups always included carnitine when group A was involved (and either B or C). Further research is necessary to gain better insight into these relationships and their potential importance for human milk composition and their potential use as indicator for maternal and infant status, genotypes and phenotypes, and other outcomes.

## Conclusions

To the best of our knowledge, this is the first reported method for human milk water-soluble choline that also includes the simultaneous analysis of six additional choline-related metabolites. The method required minimal sample volumes and preparation, allowing a high sample throughput with robust and reliable results. While not done for this report, the simple sample processing is easily transferable to a 96-well plate format, increasing the sample throughput further and allowing for additional simple and fast purification steps such as phospholipid removal, if desired. Enabling the analysis of these metabolites not only in plasma but now in milk allows for more in-depth examinations of the metabolite patterns, changes, and effects within the mother–milk–infant symbiotic relationship. While preliminary and limited, our pilot study offers additional and novel information about choline and related metabolites in human milk and their changes during lactation. More data are needed to fully understand the potential of human milk nutrients and metabolites as indicator for maternal and infant clinical outcomes.

## Data Availability Statement

The raw data supporting the conclusions of this article will be made available by the authors, without undue reservation.

## Ethics Statement

The studies involving human participants were reviewed and approved by Committees of the Municipal Secretariat of Health and Civil Defense of the State of Rio de Janeiro (protocol number 49218115.0.0000.5275), and of the Maternity School of Rio de Janeiro Federal University (protocol number 49218115.0.0000.5275). The patients/participants provided their written informed consent to participate in this study.

## Author Contributions

DH, SS-F, and LA: conceptualization and methodology. DH and NN: validation and sample analysis. DH: formal analysis, writing—original draft preparation, and writing—review and editing. GK: field study. LA and GK: funding acquisition. All authors have read and agreed to the published version of the manuscript.

## Conflict of Interest

The authors declare that the research was conducted in the absence of any commercial or financial relationships that could be construed as a potential conflict of interest.
