# Supplementary material for: High-Throughput Analysis of Water-Soluble Forms of Choline and Related Metabolites in Human Milk by UPLC-MS/MS and Its Application
Source: Front Nutr. 2021 Feb 5;7:604570. doi: 10.3389/fnut.2020.604570 (PMC7892616; doi:10.3389/fnut.2020.604570)
Supplement: Supplementary file 1 [file Table_1.DOCX]

**Supplemental Figure S1:** Chemical structures of water-soluble choline and related metabolites

|  | |
| --- | --- |
| **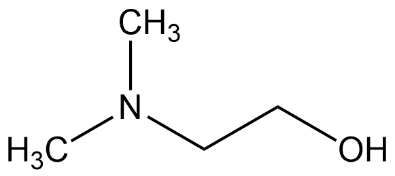Choline** | **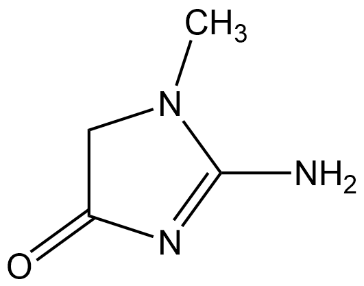Creatinine** |
| **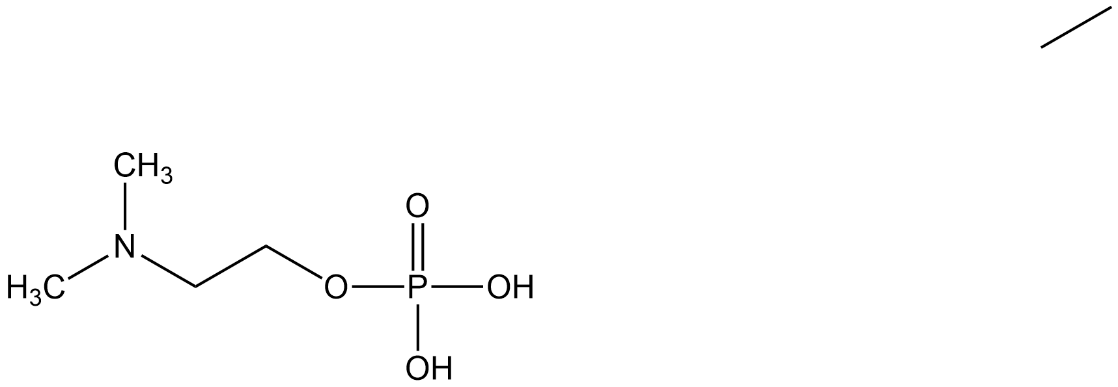Phospho-choline** | **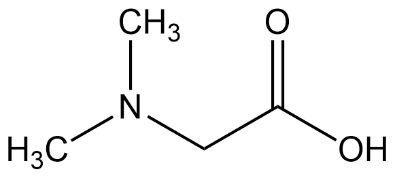Dimethylglycine (DMG)** |
| **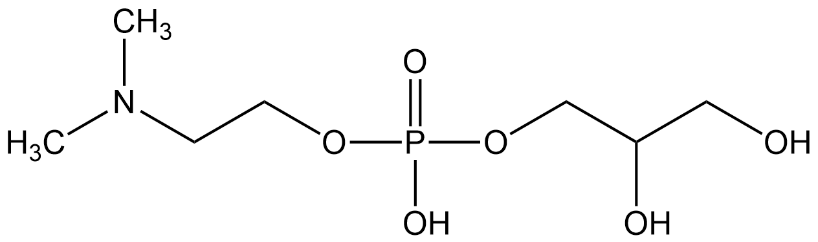Glycerophospho-choline** | **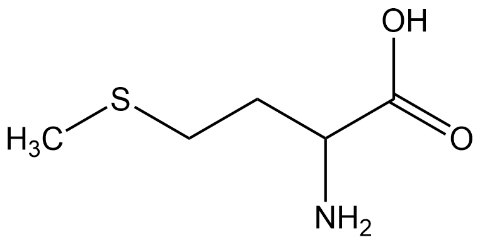Methionine** |
| **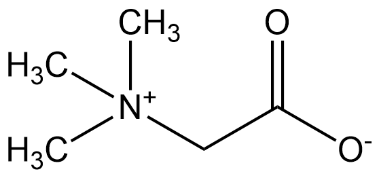Betaine** | **Trimethylamine N-oxide (TMAO)**  **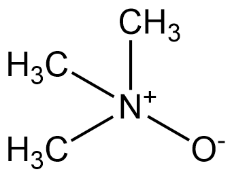** |
| **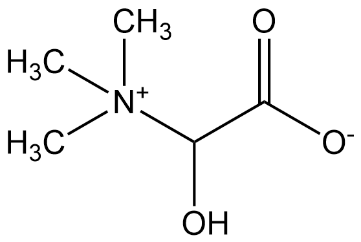Carnitine** |  |


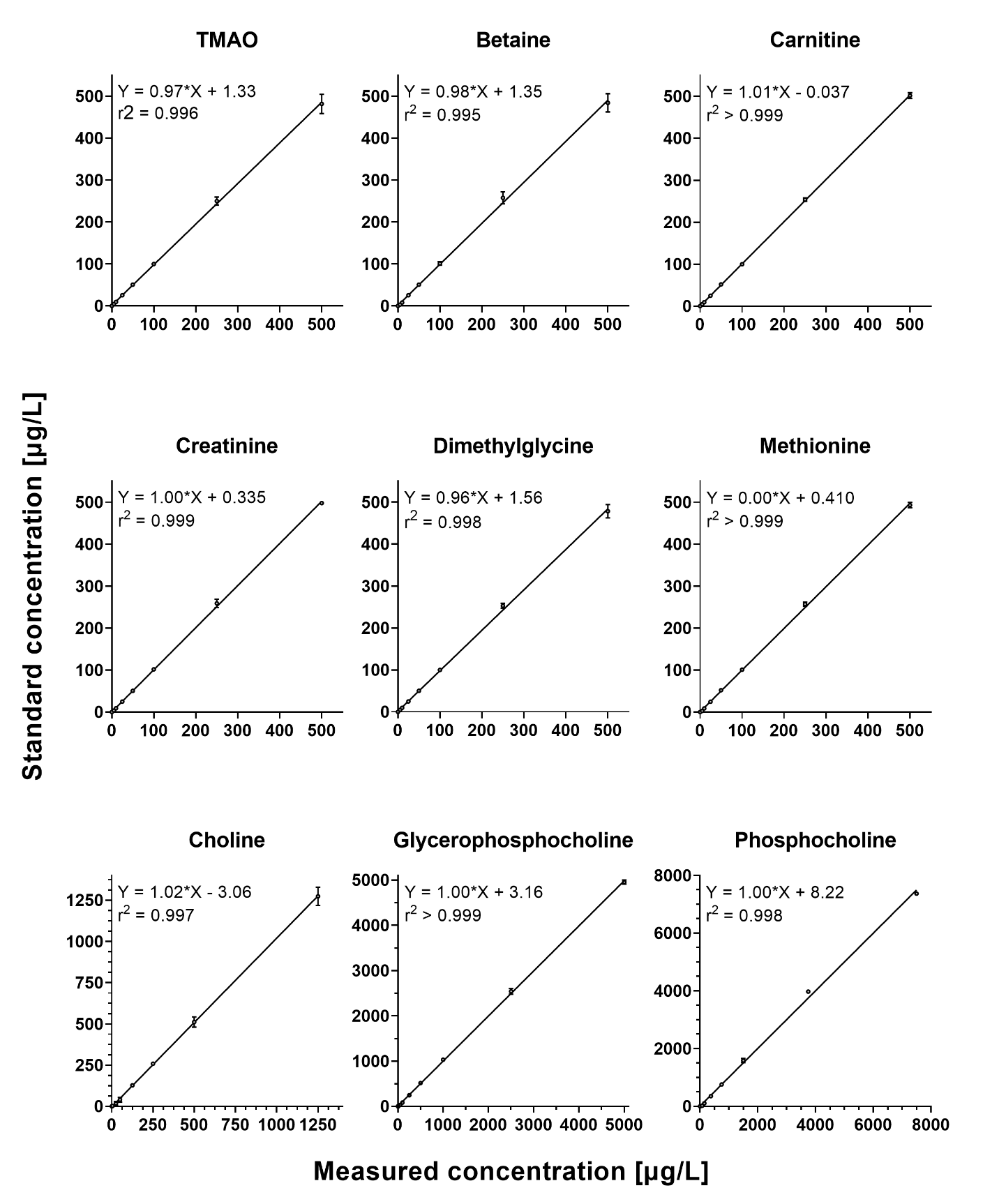
**Supplemental Figure S2:** Standard curves and trend lines of water-soluble choline and related metabolites

Standard curves are created from 8 separate standard curves over one month of analysis. If error bars are not visible their size is smaller than the symbol.
